# Primary Hypothyroidism Unleashing Severe Pericardial Effusion: Lessons Learnt From an Atypical Presentation of a Common Endocrine Condition

**DOI:** 10.7759/cureus.46947

**Published:** 2023-10-13

**Authors:** Ashutosh Kapoor, Zin Htut, Chukwuma Uduku

**Affiliations:** 1 Diabetes and Endocrinology, Chelsea and Westminster Hospital NHS Foundation Trust, London, GBR; 2 Diabetes and Endocrinology, Imperial College Healthcare NHS Trust, London, GBR

**Keywords:** cardiology manifestations, cardiology imaging, internal medicine and endocrinology, hypothyroid, severe hypothyroid states, hypothyroid pericardial effusion

## Abstract

Primary hypothyroidism is a commonly encountered endocrine disorder and can be associated with pericardial effusion and cardiac tamponade in severe cases. Early detection of hypothyroidism is key since it is a potentially treatable and reversible cause of pericardial effusions.

A 53-year-old female was admitted following a fall. The clinical history was remarkable, with symptoms of persistent tiredness and fatigue for six months. She had no known medical conditions and was not taking any regular medications. Vital signs were stable. Physical examination revealed bilateral pitting pedal oedema and a tense abdomen with shifting dullness. Cardiovascular and respiratory examinations were normal. Notably, the patient exhibited delayed relaxation of deep-tendon reflexes bilaterally at the patellar and ankle sites.

Pertinent laboratory findings showed an elevated thyroid-stimulating hormone (TSH) level of 151.69 milliunits/L, a low free thyroxine (fT4) level of <5.4 pmol/L, a haemoglobin level of 85 g/L, and a markedly high anti-thyroid peroxidase antibody level of 957.35 IU/mL. An electrocardiogram revealed a normal sinus rhythm with a low-voltage QRS complex. Chest X-ray findings indicated cardiomegaly suggestive of left heart failure. An emergent transthoracic echocardiography (TTE) demonstrated a large pericardial effusion measuring 5.4 cm posterior to the left ventricle.

The most likely aetiology in this case was severe primary hypothyroidism. She initially received intravenous liothyronine 10 micrograms every four hours, followed by oral liothyronine 5 micrograms twice a day in conjunction with levothyroxine 100 micrograms once a day. The adrenal reserve assessment was satisfactory. An urgent pericardiocentesis was performed, draining a total of 900 mL of serosanguinous fluid. Serial echocardiograms demonstrated the absence of residual effusion.

Hypothyroidism is a relatively uncommon cause of pericardial effusion. By ensuring early detection and appropriate treatment, we can optimise patient outcomes and prevent potential complications associated with untreated hypothyroidism.

## Introduction

Primary hypothyroidism is a commonly encountered endocrine disorder that can result in varying manifestations and sequelae. This condition is more prevalent in the adult female population as compared to males. The myriad of symptoms and signs can span from mild to severe. The symptoms can be so mild that, on a frequent basis, detection of hypothyroidism can be purely incidental via biochemical testing alone.

Hypothyroidism can be associated with pericardial effusion, thus causing cardiac tamponade in severe cases, which can result in a life-threatening sequela. The relationship between pericardial effusions and hypothyroidism is directly proportional to the severity of hypothyroidism and is one of its most significant complications, which can result in increased mortality [1].

In this case, we endeavour to draw attention to the importance of early detection and optimal management of hypothyroidism. This would result in improved clinical outcomes, leading to reduced hospital admissions and healthcare burden, thus improving morbidity and mortality [1,2]. Hypothyroidism is a complex endocrine disorder that can give rise to various complications, leading to increased morbidity if left untreated. Among these complications, although infrequently encountered, pericardial effusion holds particular significance. Recognising the interplay between hypothyroidism and pericardial effusion is essential for comprehensive patient management. Pericardial effusion in the context of hypothyroidism can pose a life-threatening situation due to the potential development of cardiac tamponade and subsequent haemodynamic instability. Early diagnosis and management of pericardial effusion in hypothyroidism are crucial and can serve as a life-saving measure [2].

Cardiovascular manifestations related to hypothyroidism encompass a wide range of signs and symptoms, primarily stemming from the effects of thyroid hormone deficiency. Triiodothyronine (T3), a key thyroid hormone, plays a vital role in promoting tissue oxygen consumption, enhancing the systolic contraction force, facilitating diastolic relaxation, and reducing vascular resistance [2]. The development of pericardial effusion in hypothyroidism can be attributed to increased permeability of epicardial vessels and diminished lymphatic drainage of albumin, resulting in fluid accumulation within the pericardial space [3,4]. The absence of adequate T3 levels in hypothyroidism commonly leads to the presentation of bradycardia, diastolic hypertension, and narrowed pulse pressure [5,6,7].

When overt hypothyroidism is present and other potential secondary causes have been thoroughly investigated and excluded, pericardial effusion is deemed to be secondary to the underlying thyroid-related aetiology. Timely identification of pericardial effusion and prompt initiation of appropriate interventions are vital in preventing complications such as cardiac tamponade and associated haemodynamic compromise. In this paper, we aim to shed light on the importance of early detection and management of this potentially life-threatening condition. Through a comprehensive analysis of relevant literature and clinical observations, we strive to provide valuable insights that contribute to improved outcomes for individuals experiencing this unique association between hypothyroidism and pericardial effusion.

## Case presentation

We present the case of a 53-year-old female who was admitted to our hospital via the acute medical department following a fall at a coach station. The clinical history was remarkable, with symptoms of persistent tiredness and fatigue for six months. She had no known medical conditions and was not taking any regular medications or over-the-counter drugs.

On initial assessment, the patient's vital signs were stable, with a heart rate of 66 beats per minute, blood pressure of 123/86 mmHg, and oxygen saturation of 98% on room air. Physical examination revealed bilateral pitting pedal oedema and a tense abdomen with shifting dullness. A cardiovascular examination revealed normal heart sounds without any murmurs, and the lungs were clear upon auscultation. Notably, the patient exhibited delayed relaxation of deep-tendon reflexes bilaterally at the patellar and ankle sites.

Investigations

Pertinent laboratory findings showed an elevated thyroid-stimulating hormone (TSH) level of 151.69 milliunits/L, a low free thyroxine (fT4) level of <5.4 pmol/L, a haemoglobin level of 85 g/L, and a markedly high anti-thyroid peroxidase antibody level of 957.35 IU/mL (Table 1).

**Table 1:** Biochemical investigations TSH: thyroid-stimulating hormone; fT3: free triiodothyronine; fT4: free thyroxine; WBC: white blood cells; Hb: haemoglobin; Na: sodium; K: potassium; eGFR: estimated glomerular filtration rate; CRP: C-reactive protein

	Day 1	Day 3	Day 7	Reference range
TSH	151.69	83.28	97.17	0.30-4.20 milliunit/L
fT3			<2.3	2.4-6.0 pmol/L
fT4	< 5.4	<5.4	5.4	9.0-23.0 pmol/L
Cortisol	2508	>3300	433	160-550 nmol/L
WBC	4.5	6.7	3.8	4.2-11.2 x10^9/L
Hb	85	89	87	114-150 g/L
Platelet	205	237	179	135-400x10^9/L
Neutrophil	3.6	6.1	2.9	2-7.1 x10^9/L
Na	141	139	142	133-146 mmol/L
K	3.2	3.7	4.3	3.5-5.3 mmol/L
Creatinine	107	100	67	60-125 umol/L
eGFR	>90	78	>90	
CRP	5.6	7.5	15.9	0-5 mg/L

**Figure 1:**
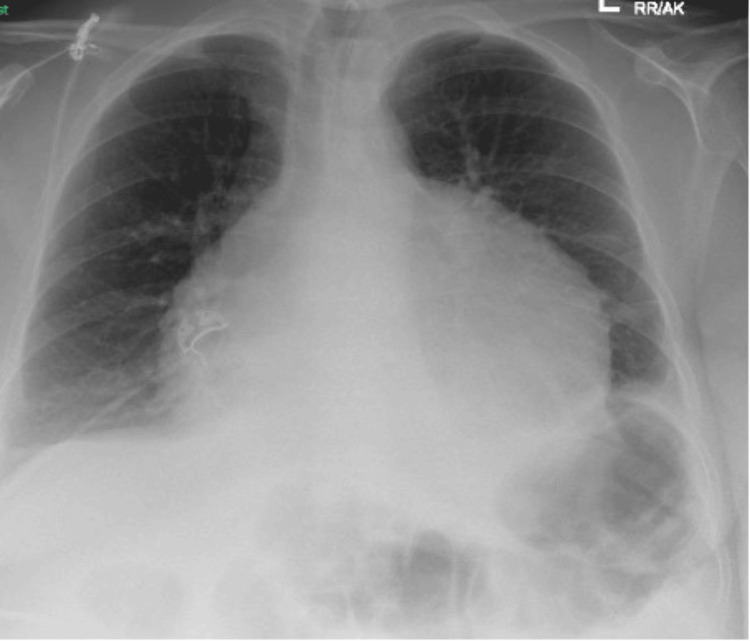
The chest X-ray shows an enlarged heart. There are small effusions at both costophrenic angles and upper lobe blood diversion, and minimal opacity at the right base and in the lingula, which suggest a degree of left heart failure.

An electrocardiogram revealed a normal sinus rhythm with a low-voltage QRS complex. Chest X-ray findings indicated cardiomegaly suggestive of left heart failure (Figure 1).

**Figure 2:**
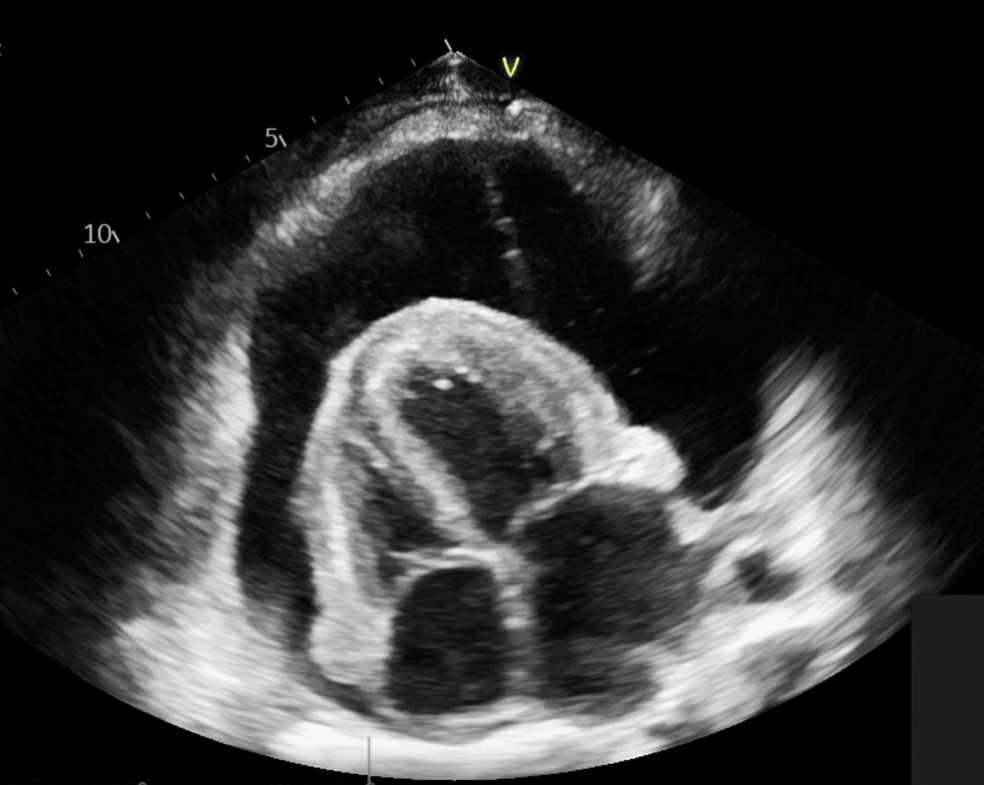
Standard echocardiographic views are used to assess a pericardial effusion. As seen in the figure above, the pericardial effusion is impeding normal myocardial contractility. The emergent transthoracic echocardiography (TTE) demonstrated a large pericardial effusion measuring 5.4 cm posterior to the left ventricle.

An emergent transthoracic echocardiography (TTE) demonstrated a large pericardial effusion measuring 5.4 cm posterior to the left ventricle (Figure 2).

Additionally, there was early diastolic collapse of the right ventricle and systolic collapse of the right atrium. The presence of respiratory variations in mitral and tricuspid inflow velocities was consistent with tamponade features. The ultrasound of the abdomen revealed a large volume of ascites. Furthermore, a computed tomography (CT) scan of the head, performed due to the fall, revealed a left orbital blowout fracture with an infraorbital hematoma managed conservatively.

Treatment

Based on the clinical presentation and investigation findings, the most likely aetiology in this case was severe primary hypothyroidism, thus resulting in a massive pericardial effusion. Other secondary causes, such as nephrotic syndrome, liver dysfunction, and hypoalbuminemia, were thoroughly ruled out by a normal urine dip, normal albumin levels, and normal liver function tests. The patient initially received intravenous liothyronine 10 micrograms every four hours, followed by oral liothyronine 5 micrograms twice a day in conjunction with levothyroxine 100 micrograms once a day. In view of delirium initially, she was refusing oral medications, therefore, to ensure patient safety, treatment was commenced on IV liothyronine (multiple doses/day due to short half-life) and also covered with IV hydrocortisone. The cortisol levels in the biochemical investigations on days one and two are actually post-hydrocortisone.

An urgent pericardiocentesis was performed, draining a total of 900 mL of serosanguinous fluid. Analysis of the pericardial fluid did not reveal any remarkable findings. Serial echocardiograms demonstrated the absence of residual effusion or re-accumulation. There was no evidence of heart failure, and the ejection fraction was within normal limits. There was a concurrent improvement in ascites following the restoration and normalisation of thyroid function.

Outcome and follow-up

The patient remained hemodynamically stable throughout her hospital stay and was discharged with instructions to adhere to thyroid supplementation. Regular follow-up appointments were scheduled to monitor her progress and adjust the treatment regimen depending on serial thyroid function tests. Thyroid function monitoring with primary care has shown a drastic improvement, and she has had no further hospital admissions, along with clinical stability. An endocrinology follow-up is currently in the pipeline.

## Discussion

Given the intricate and challenging nature of the presentation, as well as the diagnostic conundrum posed by this association, engaging in a collaborative discussion within a multi-disciplinary team (MDT) setting is strongly recommended. The involvement of specialists from both endocrinology and cardiology is essential to ensure a comprehensive evaluation and safe management of the patient [1]. Endocrinology opinions are sought frequently; however, delineating the underlying aetiology in the first instance remains a challenge.

Although hypothyroidism is not commonly identified as an underlying cause of pericardial effusions, it is important to broaden our diagnostic considerations [8]. Endocrine causes should not be overlooked, as they may contribute to the development of pericardial effusions, even if they are considered rare. Including experts from endocrinology in the MDT discussion allows for a thorough exploration of potential underlying endocrine disorders that could be contributing to the patient's condition.

It is crucial to keep in mind that not all patients will present with the typical, classical features of hypothyroidism. Therefore, maintaining a low threshold for assessing hypothyroidism is crucial, especially when a new pericardial effusion is observed [5]. By being vigilant and proactive in evaluating thyroid function, we can avoid overlooking this potentially significant underlying aetiology.

Cardiovascular manifestations related to hypothyroidism encompass a wide range of signs and symptoms, primarily stemming from the effects of thyroid hormone deficiency. Triiodothyronine (T3), a key thyroid hormone, plays a vital role in promoting tissue oxygen consumption, enhancing the systolic contraction force, facilitating diastolic relaxation, and reducing vascular resistance. The development of pericardial effusion in hypothyroidism can be attributed to increased permeability of epicardial vessels and diminished lymphatic drainage of albumin, resulting in fluid accumulation within the pericardial space. The absence of adequate T3 levels in hypothyroidism commonly leads to the presentation of bradycardia, diastolic hypertension, and narrowed pulse pressure.

By engaging in a collaborative MDT discussion that incorporates insights from both endocrinology and cardiology, we can ensure a comprehensive evaluation, accurate diagnosis, and optimal management of the patient's condition. This multidimensional approach will contribute to the delivery of safe and effective care, addressing both the pericardial effusion and any potential endocrine abnormalities.

To conclude, hypothyroidism is a relatively uncommon cause of pericardial effusion, which can contribute to its underestimation in clinical practice. However, it is important to recognise and manage hypothyroidism promptly, as it is a potentially reversible condition that typically improves with the administration of thyroid hormones. It is crucial to emphasise the need for close follow-up in such cases, as it may take several months for symptoms to resolve and for the pericardial effusion to subside following levothyroxine replacement therapy. By ensuring early detection and appropriate treatment, we can optimise patient outcomes and prevent potential complications associated with untreated hypothyroidism [2].

## Conclusions

Pericardial effusion, although rare, can be a potential complication of primary hypothyroidism. Hypothyroidism should be considered a differential diagnosis in patients presenting with unexplained pericardial effusion, especially when other secondary causes have been excluded. Vigilant monitoring and thorough assessment are required in patients with hypothyroidism to detect the presence of pericardial effusion, as it can lead to life-threatening complications such as cardiac tamponade. Prompt diagnosis and initiation of appropriate management, including thyroid hormone replacement therapy, are crucial in resolving the pericardial effusion associated with hypothyroidism. Increased awareness amongst healthcare providers about the potential association between primary hypothyroidism and pericardial effusion can lead to earlier recognition and improved patient outcomes.

Pericardial effusion secondary to primary hypothyroidism is usually reversible in nature, so optimal management is of paramount importance. By utilising this case, we would like to focus on the importance of early detection and inculcating a holistic approach during the assessment of such medical presentations.
